# 

**Published:** 1870-11

**Authors:** 


*See* W. B. Keen & Cooke's *New Classified Price List of Medical Books, commencing next page.*

